# Fatty Acid Oxidation Changes and the Correlation with Oxidative Stress in Different Preeclampsia-Like Mouse Models

**DOI:** 10.1371/journal.pone.0109554

**Published:** 2014-10-10

**Authors:** Xiaoyan Ding, Zi Yang, Yiwei Han, Huan Yu

**Affiliations:** Department of Obstetrics and Gynecology, Peking University Third Hospital, Beijing, PR China; Clermont Université, France

## Abstract

**Background:**

Long-chain 3-hydroxyacyl-CoA dehydrogenase (LCHAD) expression is decreased in placenta of some cases of preeclampsia (PE) which may result in free fatty acid (FFA) increased. High FFA level will induce oxidative stress, so abnormal long-chain fatty acid-oxidation may participate in the pathogenesis of PE through oxidative stress pathway.

**Methods:**

PE-like groups were ApoC3 transgenic mice with abnormal fatty acid metabolism, classical PE-like models with injection of Nw-nitro-L-arginine-methyl ester (L-NA) or lipopolysaccharide (LPS) and the antiphospholipid syndrome (APS) mouse model with β2GPI injection (ApoC3+NS, ApoC3+L-NA, L-NA, LPS and β2GPI groups). The control group was wild-type mice with normal saline injection. Except for β2GPI mice, the other mice were subdivided into pre-implantation (Pre) and mid-pregnancy (Mid) subgroups by injection time.

**Results:**

All PE-like groups showed hypertension and proteinuria except ApoC3+NS mice only showed hypertension. Serum FFA levels increased significantly except in LPS group compared to controls (P<0.05). LCHAD mRNA and protein expression in the liver and placenta was significantly higher for ApoC3+NS, ApoC3+L-NA and β2GPI mice and lower for L-NA mice than controls (P<0.05) but did not differ between LPS mice and controls. P47phox mRNA and protein expression in the liver significantly increased in all PE-like groups except LPS group, while P47phox expression in the placenta only significantly increased in L-NA and β2GPI groups.

**Conclusions:**

Abnormal long-chain fatty acid-oxidation may play a different role in different PE-like models and in some cases participate in the pathogenesis of PE through oxidative stress pathway.

## Introduction

Preeclampsia (PE) affects 5% to 8% of pregnancies and has severe consequences for both the mother and fetus [1]. The pathogenesis of PE remains elusive. Long-chain 3-hydroxyacyl-CoA dehydrogenase (LCHAD) deficiency has been found associated with complications of pregnancy such as hemolysis, elevated liver enzymes, and low platelet syndrome (HELLP) and acute fatty liver of pregnancy [2]–[4]. We and other researchers found decreased LCHAD expression in some cases of PE without LCHAD deficiency and the expression of LCHAD significantly differed between early-onset severe PE and late-onset PE [5],[6]. The heterogeneity of PE has been gradually realized by study of different etiological factors between early- and late-onset PE [7],[8]. However, the role of long-chain fatty acid-oxidation (FAO) disorders in different pathogenesis of PE is unclear and deserves further study.

The last three steps of FAO are catalyzed by the trifunctional protein (MTP); LCHAD is located on the α-subunit of MTP [9]. LCHAD deficiency leads to long-chain FAO disorders. However, some PE patients without LCHAD deficiency also show long-chain FAO disorders. Maternal plasma from PE patients could result in lipid droplet accumulation in cultured human umbilical vein endothelial cells and cause a significant decrease in mitochondrial dehydrogenase activity [10]. In animal experiments, we found significant pathological changes in placentas of Nw-nitro-L-arginine-methyl ester (L-NAME; L-NA)-established PE-like mice in early and mid-gestational stages as well as decreased LCHAD expression [11]. An *in vitro* cell culture study of placental trophoblasts showed that LCHAD mRNA and protein expression significantly decreased in early-onset severe PE and HELLP syndrome as compared with late-onset severe PE [12]. These findings indicate that disorders of long-chain FAO induced by decreased LCHAD expression may play an important role in some patients with early-onset PE.

Yet not all patients with PE have FAO disorders. Previously, we found no significant difference in LCHAD protein expression between late-onset PE and controls [2]. No change in triglycerides (TG) level was found in a PE-like mouse model of reduced uterine perfusion pressure [13]. Therefore, different onset times and different subtypes of PE may exhibit different FAO disorders.

The mechanism about how FAO disorders result in PE is unclear. FAO disorders can cause serum FFA increase and high FFA level will activate oxidative stress response. There have been many researches about the effects of oxidative stress on endothelial injury in the pathogenesis of preeclampsia. Reactive oxygen species generated during oxidative stress attack the phospholipids of cell membranes and react with polyunsaturated fatty acids to form lipid peroxides resulting in cellular injury [14]. So we speculate that abnormal FAO may induce PE through oxidative stress pathway.

In the present study, we established classical PE-like models by L-NA and lipopolysaccharide (LPS) injection and used ApoC3 transgenic mice with abnormal fatty acid metabolism and an APS mouse model with underlying maternal disease to establish PE-like models induced by different factors. Also we chose two time points, pre-implantation and mid-gestation, to establish PE-like models induced by different times. Mid-gestation is classical time to establish PE models. We chose pre-implantation time to investigate the effects of adverse factors on the placenta before it began to form. We used this multifactorial and different time research platform to investigate the role of LCHAD and its relationship with oxidative stress in the pathogenesis of different subtypes of PE.

## Materials And Methods

### Ethics Statement

The animal experiment was approved by the Animal Care Committee and Medical Ethics Committee of Peking University (permit number: LA2012-8) and procedures were conducted according to its guidelines. All surgery was performed under anesthesia, and all efforts were made to minimize suffering.

### Establishment And Identification Of Animal Models

C57BL/6J mice were from the Department of Laboratory Animal Science, Peking University, and C57BL/6J mice with transgenic overexpression of apoC3 were supported by the Institute of Cardiovascular Sciences, Peking University Health Science Center. We housed 8- to 10-week-old virgin female and 10- to 14-week-old male mice under controlled conditions and fed them standard mouse chow with water available *ad libitum*. The mice were mated at a ratio of 2∶1 females to males and females were inspected daily for vaginal plugs, designated as day 1 of pregnancy.

Mice were randomly divided into control, ApoC3+NS, ApoC3+L-NA, L-NA, LPS and β2GPI groups. Except for the β2GPI group, the other groups were subdivided into pre-implantation (Pre) and mid-gestation (Mid) subgroups according to injection time (n = 10 per group). Transgenic mice in ApoC3+L-NA and wild-type mice in L-NA group received a daily subcutaneous injection with L-NA (Sigma, USA), 50 mg/kg/d, [15],[16] from day 3 (Pre) or 11 (Mid) to day 17 of pregnancy. For LPS mice, wild-type mice received a single injection with an ultra-low dose of LPS (1 µg/kg body weight, Sigma) on day 3 or 11 of pregnancy [17],[18]. β2GPI mice had a weekly subcutaneous injection with complete Freund's adjuvant-dissolved human β2GPI (25 µg per mouse, Sigma) in the back 3 weeks before mating and incomplete Freund's adjuvant-dissolved β2GPI 2 weeks and 1 week before mating [19]. Wild-type mice in the control group and transgenic mice in the ApoC3+NS group were injected daily with physiological saline from day 3 or 11 of pregnancy.

From day 2 of gestation, a CODA non-invasive tail-cuff acquisition system (Kent Scientific Corp., USA) was used to measure blood pressure every 2 days. The mice were placed in standard metabolism cages on day 17 of pregnancy and 24-hr urine was collected. The detection of urinary protein involved a protein assay kit (Bio-Rad, USA).

### Sample Collection

All mice were anesthetized with 10% chloral hydrate (3 ml/kg) on day 18 of pregnancy. Blood samples, taken from the retro-orbital plexus, were centrifuged and serum was collected. Cesarean section was performed, and the number of live births, absorption number and fetal and placental wet weight were recorded. Liver and placenta tissues were collected; some were embedded with Optimal Cutting Temperature compound for Oil-red O staining, some were fixed in formalin for immunohistochemistry, and the remainders were frozen at −80°C for mRNA and protein detection. Finally the mice were terminated by cervical dislocation.

### Histological Analysis

Frozen mouse liver and placenta tissues were sliced into 10-µm sections, stained with Oil-red O (GenMed Scientifics, USA). Sections were photographed under an optical microscope (Nikon, Canada) and stained area was assessed by use of NIS-Elements BR 3.2 software.

### Quantitative Real-Time Pcr

TRIzol reagent (Sigma, USA) was used to extract total RNA from the liver and placenta. Total RNA, 1 µg, was reverse-transcribed to cDNA by use of the Revert Aid First Strand cDNA Synthesis Kit (Thermo, USA). The real-time quantitative PCR reaction system involved SYBR Select Master Mix reagent (Invitrogen Life Technologies, USA) and PCR amplification involved a 7500 Real-Time PCR System (Life Technology, USA). Primer synthesis was completed by Sangon Biotech (Shanghai) with the primer sequences for LCHAD, forward, 5′-TGCATTTGCCGCAGCTTTAC-3′, and reverse, 5′-GTTGGCCCAGATTTCGTTCA-3′; p47phox, forward, 5′-ACACCTTCATTCGCCATATTGC-3′, and reverse, 5′-CCTGCCACTTAACCAGGAACA-3′; and GAPDH (as an internal control) forward, 5′-TGATGACATCAAGAAGGTGGTGAAG-3′, and reverse, 5′-TCCTTGGAGGCCATGTAGGCCAT-3′. PCR conditions were 94°C for 2 min; 55-60°C for 30 s and 72°C for 1 min, 40 cycles.

### Immunohistochemistry

Sections were deparaffinized and antigen retrieval was performed with EDTA (PH 9.0) at 98°C for 20 min. After blocking for endogenous peroxidase with 3% H2O2, sections were incubated with antibodies to LCHAD (Abcam, UK; 1∶400) at 4°C overnight and with appropriate secondary antibodies (OriGene, Beijing) at room temperature for 30 min. Diaminobenzidine was used as a chromogen. Sections were counterstained with hematoxylin. Immunohistochemical images were assessed by use of Image-Pro Plus 6.0, and the integral optical density (IOD) of each photograph was collected.

### Western Blot Analysis

Protein was extracted from liver and placenta tissues by use of RIPA lysis buffer (cwbiotech, China) with prior addition of protease inhibitors (Pierce, USA). An equal amount of protein sample was used for electrophoresis in 10% polyacrylamide gel and transferred onto a 0.45-µm PVDF membrane (Millipore, USA), which was blocked with 5% milk (BD, USA) at room temperature for 1 hr, then incubated with primary antibodies rabbit anti-mouse LCHAD (Abcam, UK; 1∶500), rabbit anti-mouse p47phox (Santa Cruz, USA, 1∶500) and rabbit anti-mouse β-actin (Cell Signaling, USA; 1∶1000) at 4°C overnight. Membranes were washed at room temperature for 5 min×5 times, then horseradish peroxidase-conjugated secondary antibody was added (1∶10000, Thermo, USA) for incubation at room temperature for 1 hr, then washed again for 5 min×5 times. The KODAK gel logic 4000MM PRO imaging system (Kodak, USA) was used for scanning and detection of bands. The relative expression of the target protein to β-actin was calculated.

### Statistical Analysis

Quantitative data are expressed as mean±SD. One-way ANOVA followed by Student-Newman-Keuls or Games-Howell test was used for comparing multiple groups. Qualitative data were compared by chi-square test. Pearson correlational analysis was used for comparing results of FFA levels, LCHAD and p47phox expression. P<0.05 was considered statistically significant.

## Results

### Confirmation Of Pe Models

**Figure 1:**
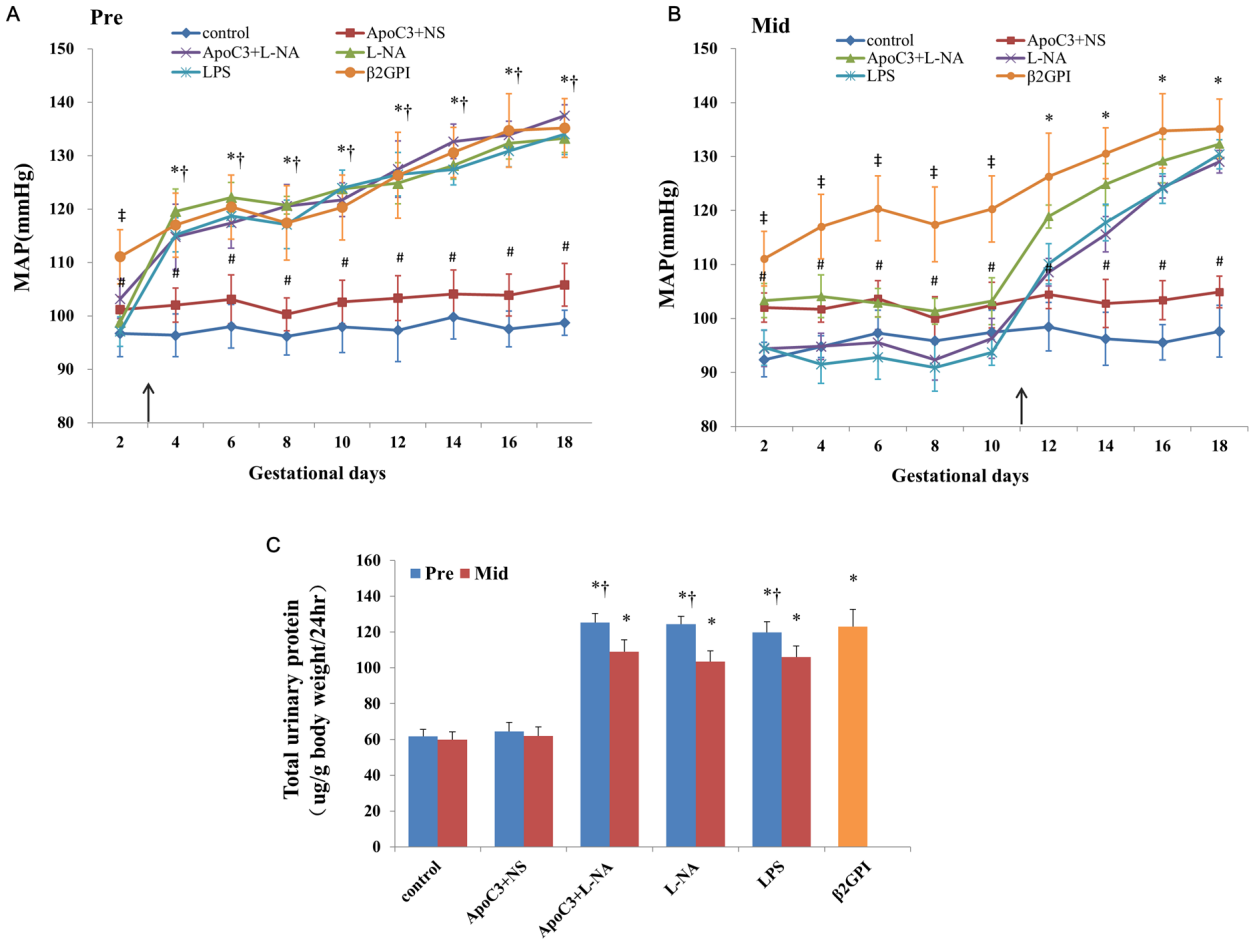
Mean arterial blood pressure throughout pregnancy in Pre (A) and Mid subgroups (B) and 24-hr urinary protein levels (C). The arrow indicates time of injection except for the β2GPI group. #P<0.05 compared with control. *p<0.05 compared with control and ApoC3+NS. ‡p<0.05 compared with control and other experiment groups. †P<0.05, compared with corresponding Mid-gestation group. Data are mean±SD, n = 10. Pre, pre-implantation. Mid, Mid-gestation.

Mice in ApoC3+NS only exhibited mild gestational hypertension, but after L-NA injection, the mean arterial pressure (MAP) and urinary protein levels were higher for ApoC3+L-NA than ApoC3+NS mice (P<0.05), with PE-like symptoms (Fig. 1A,B). Other groups also showed PE-like symptoms compared with controls. MAP was elevated from day 2 of pregnancy in β2GPI mice and from the second day after injection in ApoC3+L-NA, L-NA and LPS mice. MAP in all PE-like groups except ApoC3+NS group increased with the gestational time. MAP was higher for the Pre than Mid subgroup of ApoC3+L-NA, L-NA and LPS mice (P<0.05).

Urine protein level did not differ between controls and ApoC3+NS mice but was higher for other PE-like groups than controls, with no significant difference among these PE-like groups. Urine protein level was higher for the Pre than Mid subgroup for ApoC3+L-NA, L-NA and LPS mice (P<0.05; Fig. 1C).

### Feto-Placental Outcomes

Except for the ApoC3+NS group, mice in all PE-like groups had adverse pregnancy outcomes, including high fetal absorption rate and low fetal and placental weight, as compared with controls (Table 1).

**Table 1:** Feto-placental outcomes in all treatment groups.

Groups	Live fetuses (%)		Absorbed fetuses (%)		Fetal weight (g)		Placental weight (mg)	
	Pre	Mid	Pre	Mid	Pre	Mid	Pre	Mid
**Control**	89 (95.7)	87 (96.7)	4 (4.3)	3 (3.3)	0.82±0.08	0.84±0.06	94.4±6.0	90.4±5.9
**ApoC3+NS**	82 (95.3)	85 (96.6)	4 (4.7)	3 (3.4)	0.80±0.09	0.81±0.08	97.2±5.9	94.2±4.0
**ApoC3+L-NA**	79 (86.8)^a^	77 (89.5)^a^	12 (13.2)^a^	9 (10.4)^a^	0.63±0.07^a^	0.67±0.07^a^	75.9±5.2^a^	78.2±4.9^a^
**L-NA**	75 (87.2)^a^	75 (90.5)^a^	11 (12.8)^a^	8 (9.5)^a^	0.71±0.08^a^	0.73±0.06^a^	70.0±7.8^a^	80.7±5.6^a^^,^ b
**LPS**	77 (86.5)^a^	71 (87.7)^a^	12 (13.5)^a^	10 (12.3)^a^	0.67±0.07^a^	0.72±0.06^a^	73.3±3.6^a^	79.4±4.5^a^^,^ b
**β2GPI**	71 (86.6)^a^		11 (13.4)^a^		0.71±0.06^a^		77.1±6.9^a^	

Data are mean±SD or number (%) and n = 10 per group.

a P<0.05 compared with Control and ApoC3+NS.

b P<0.05 compared with corresponding Pre group.

Fetal absorption rate and fetus weight did not differ among Pre subgroups and corresponding Mid subgroups of PE-like groups. Placenta weight was significantly lower in the Pre than Mid subgroup for L-NA mice and LPS mice (P<0.05) (Table 1).

### Liver And Placenta Fat Staining

**Figure 2:**
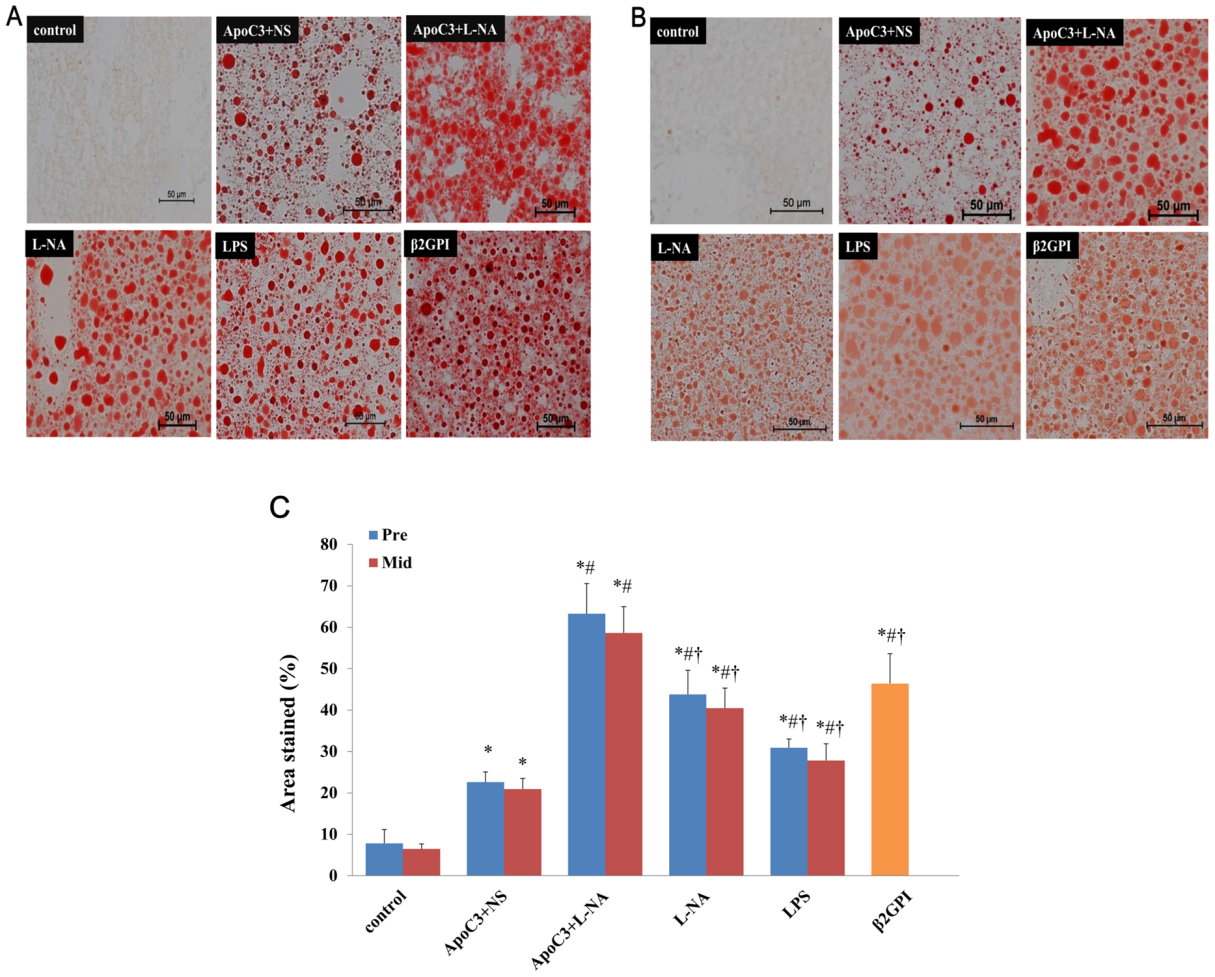
Lipid deposition in liver tissues with Oil-red O staining at pre-implantation (A) and mid-gestational age (B). (Original magnification ×200, scale bars 50 µm) (C): Percentage of area stained in all groups. *p<0.05 compared with control. #P<0.05 compared with ApoC3+NS. †p<0.05 compared with ApoC3+L-NA. Data are mean±SD, n = 10. Pre, pre-implantation. Mid, Mid-gestation.

Fat staining showed aggregation of lipid droplets in mouse liver tissues was greater in the PE-like groups than controls (Fig. 2A,B). The percentage of area stained was higher for ApoC3+NS, ApoC3+L-NA, L-NA, LPS and β2GPI mice than controls and the percentage for ApoC3+L-NA mice was significantly higher than for the other PE-like groups (P<0.05). Pre and corresponding Mid subgroups did not differ in fat staining (Fig. 2C).

**Figure 3:**
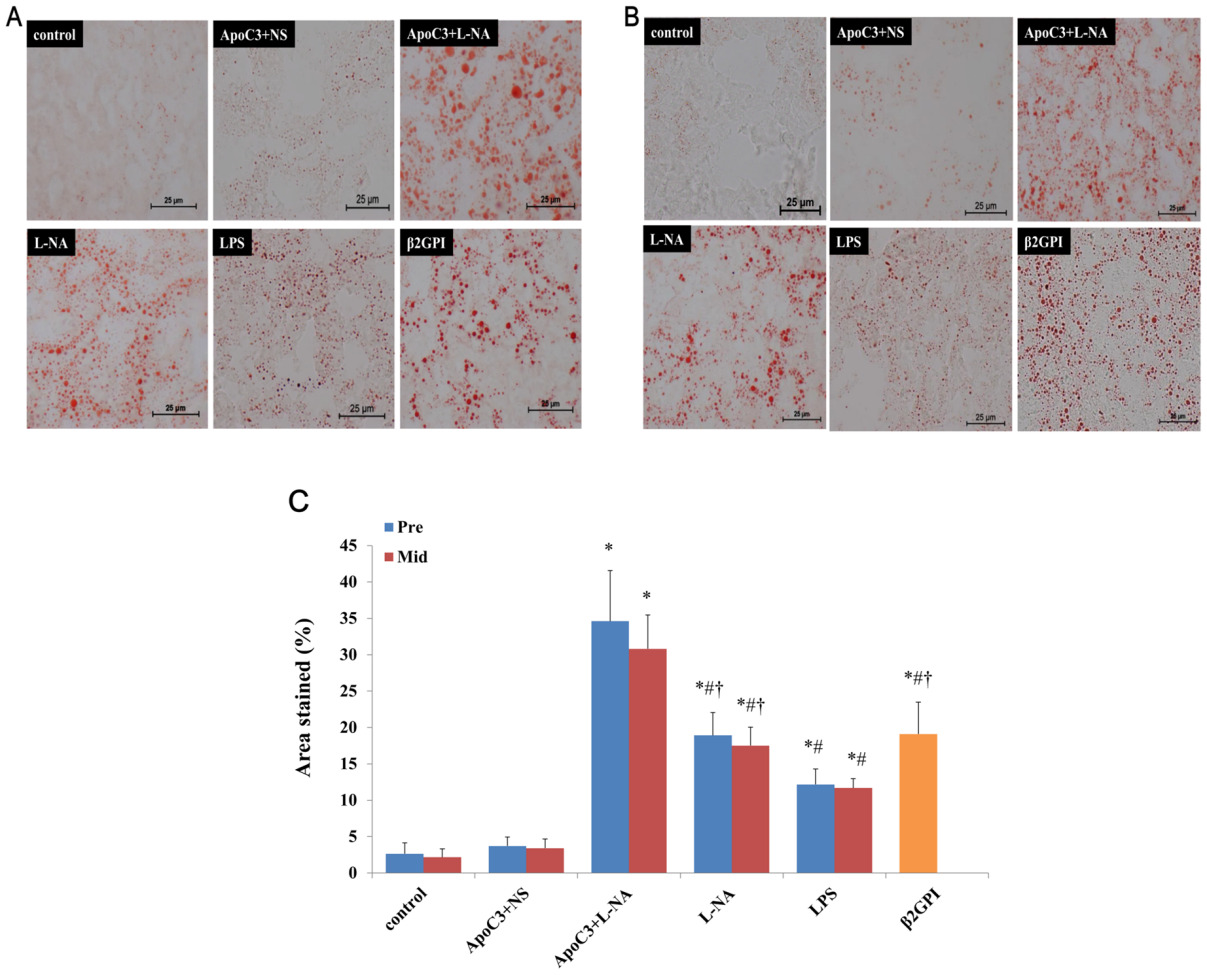
Lipid deposition in placenta tissues with Oil-red O staining at pre-implantation (A) and mid-gestational age (B). (Original magnification ×400, scale bars 25 µm) (C): Percentage of area stained in all groups. *p<0.05 compared with control and ApoC3+NS. #P<0.05 compared with ApoC3+L-NA. †p<0.05 compared with LPS. Data are mean±SD, n = 10. Pre, pre-implantation. Mid, Mid-gestation.

Placenta tissues of control and ApoC3+NS groups showed almost no formation of lipid droplets, but other PE-like groups showed a large amount of lipid droplets aggregated in placenta tissues (Fig. 3A,B). The percentage of area stained was higher for ApoC3+L-NA, L-NA, LPS and β2GPI mice than controls (P<0.05), with no difference between ApoC3+NS and controls. The percentage of area stained was highest for ApoC3+L-NA mice among all groups and was lower for LPS than ApoC3+L-NA, L-NA and β2GPI groups (P<0.05). Formation of lipid droplets did not differ between Pre and corresponding Mid subgroups (Fig. 3C).

### Ffa Levels

**Figure 4:**
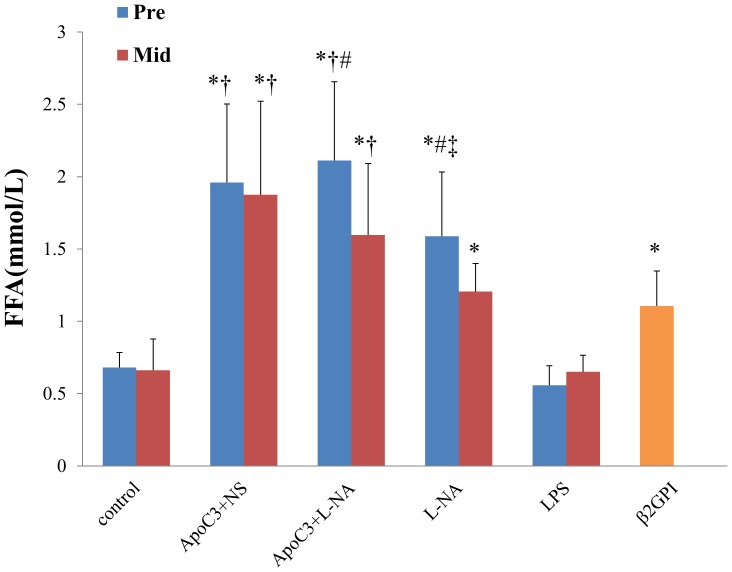
Serum FFA levels in all groups. *p<0.05 compared with control. †p<0.05 compared with L-NA and β2GPI. ‡p<0.05 compared withβ2GPI. #P<0.05 compared with the corresponding Mid group. Data are mean±SD, n = 10. Pre, pre-implantation. Mid, Mid-gestation.

FFA levels in all PE-like groups were significantly higher than the corresponding control group except the LPS group (P<0.05). FFA levels in ApoC3+NS and ApoC3+L-NA groups were significantly higher than L-NA and β2GPI groups (P<0.05). In Pre groups FFA levels in the L-NA group were significantly higher than the β2GPI group (P<0.05). For ApoC3+L-NA and L-NA groups, FFA levels in Pre groups were significantly higher than the corresponding Mid groups (P<0.05) (Fig. 4).

### Lchad Mrna Expression In Liver And Placenta Tissues

**Figure 5:**
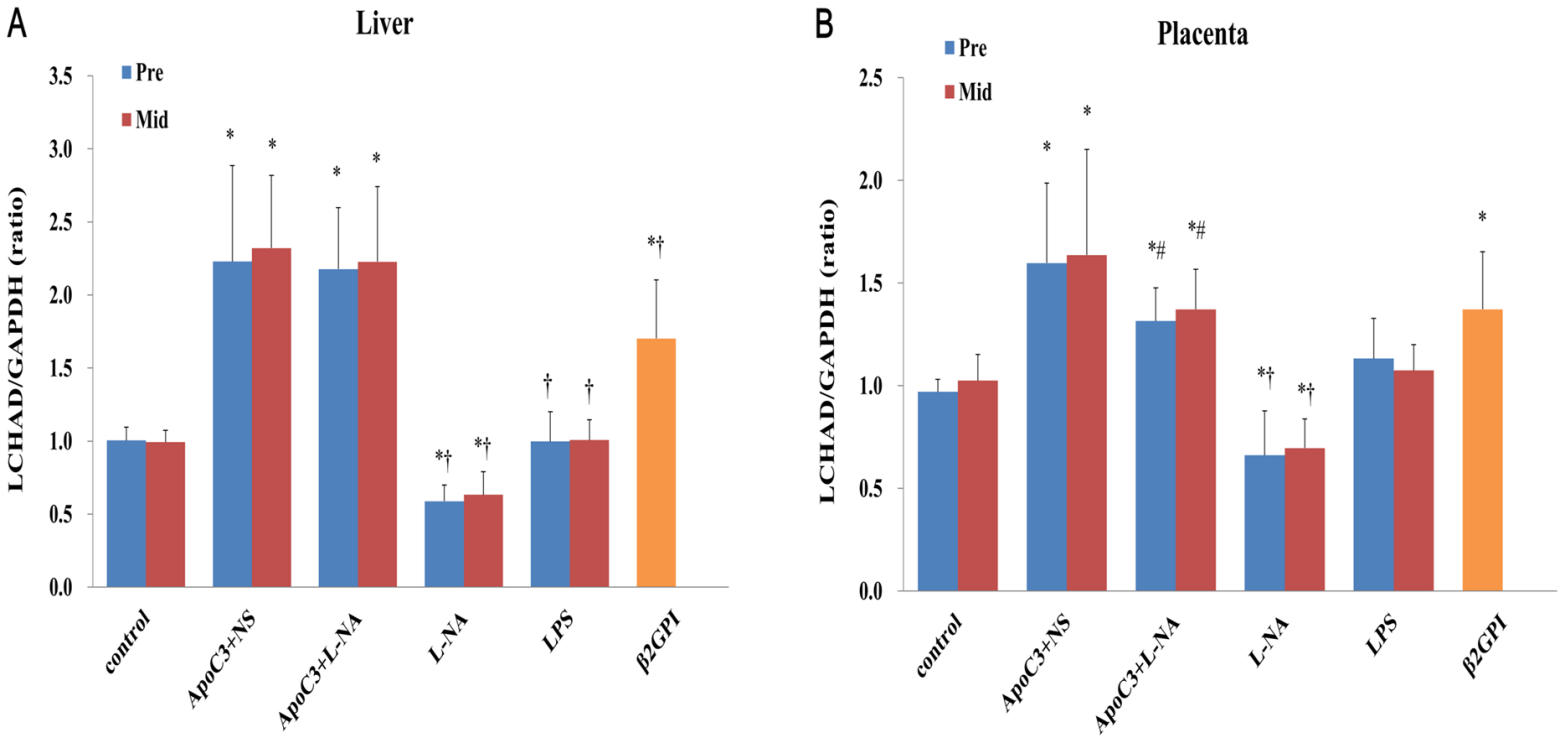
Quantified expression of long-chain 3-hydroxyacyl-CoA dehydrogenase (LCHAD) mRNA level in liver (A) and placenta (B). *P<0.05 compared with control. †P<0.05 compared with ApoC3+L-NA. #P<0.05 compared with ApoC3+NS. Data are mean±SD, n = 10. Pre, pre-implantation. Mid, Mid-gestation.

Pre and corresponding Mid subgroups did not differ in LCHAD mRNA expression (Fig. 5A,B). LCHAD mRNA expression was higher in both liver and placenta of ApoC3+NS, ApoC3+L-NA, and β2GPI mice than controls (P<0.05) and was lower in L-NA mice than controls (P<0.05), with no significant difference in LPS mice. LCHAD mRNA level in liver was higher for ApoC3+L-NA than other PE-like groups (P<0.05), with no significant difference compared to ApoC3+NS mice (Fig. 5A). LCHAD mRNA expression in placenta was lower for ApoC3+L-NA than ApoC3+NS mice (P<0.05) (Fig. 5B).

### Lchad Protein Expression In Placenta And Liver

**Figure 6:**
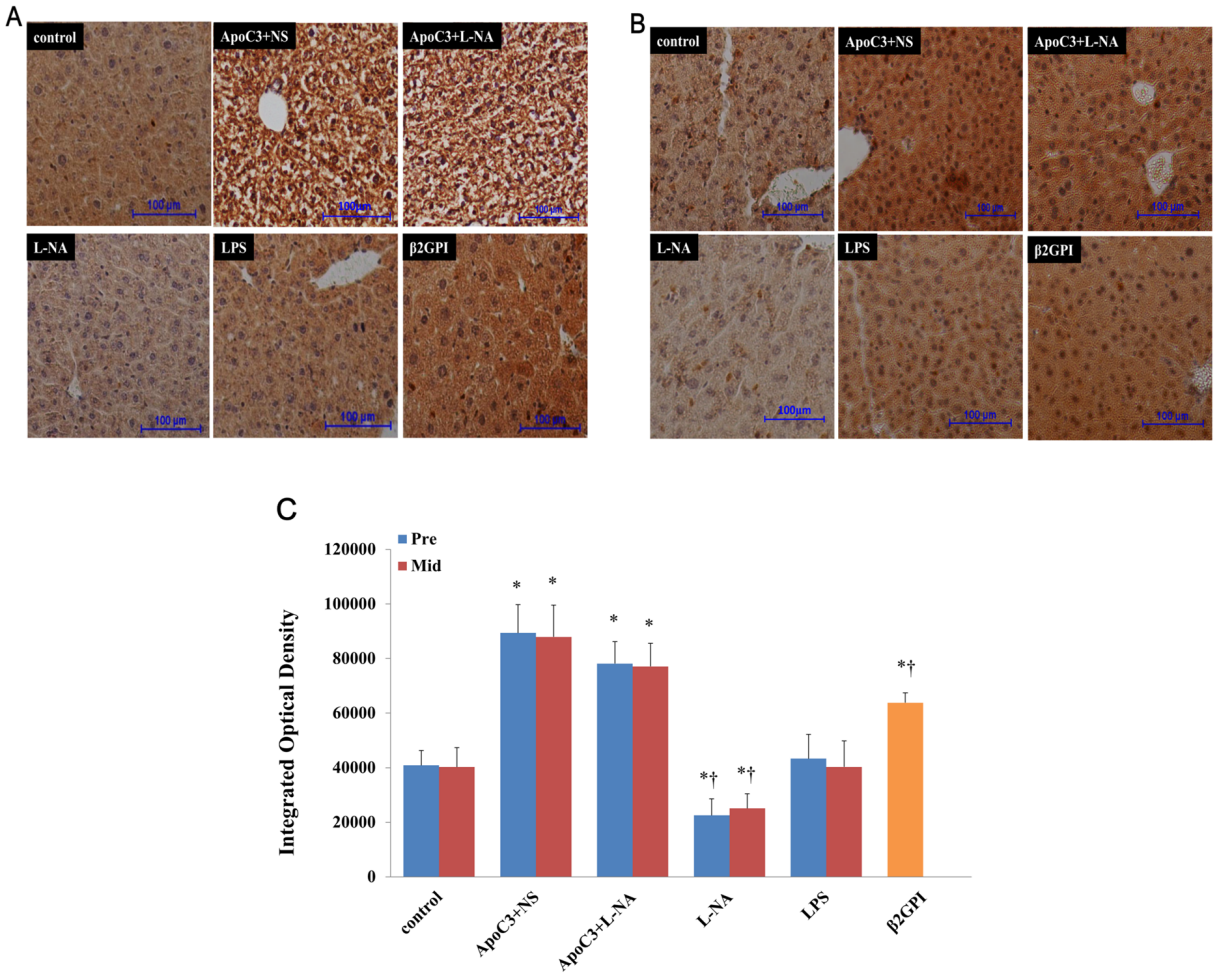
Representative immunohistochemical staining of LCHAD in mouse liver at pre-implantation (A) and mid-gestational age (B). (Original magnification ×100, scale bars 100 µm) (C): Integrated optical density (IOD) of liver immunohistochemical images in all groups. *P<0.05 compared with control. †P<0.05 compared with ApoC3+L-NA. Data are mean±SD, n = 10. Pre, pre-implantation. Mid, Mid-gestation.

**Figure 7:**
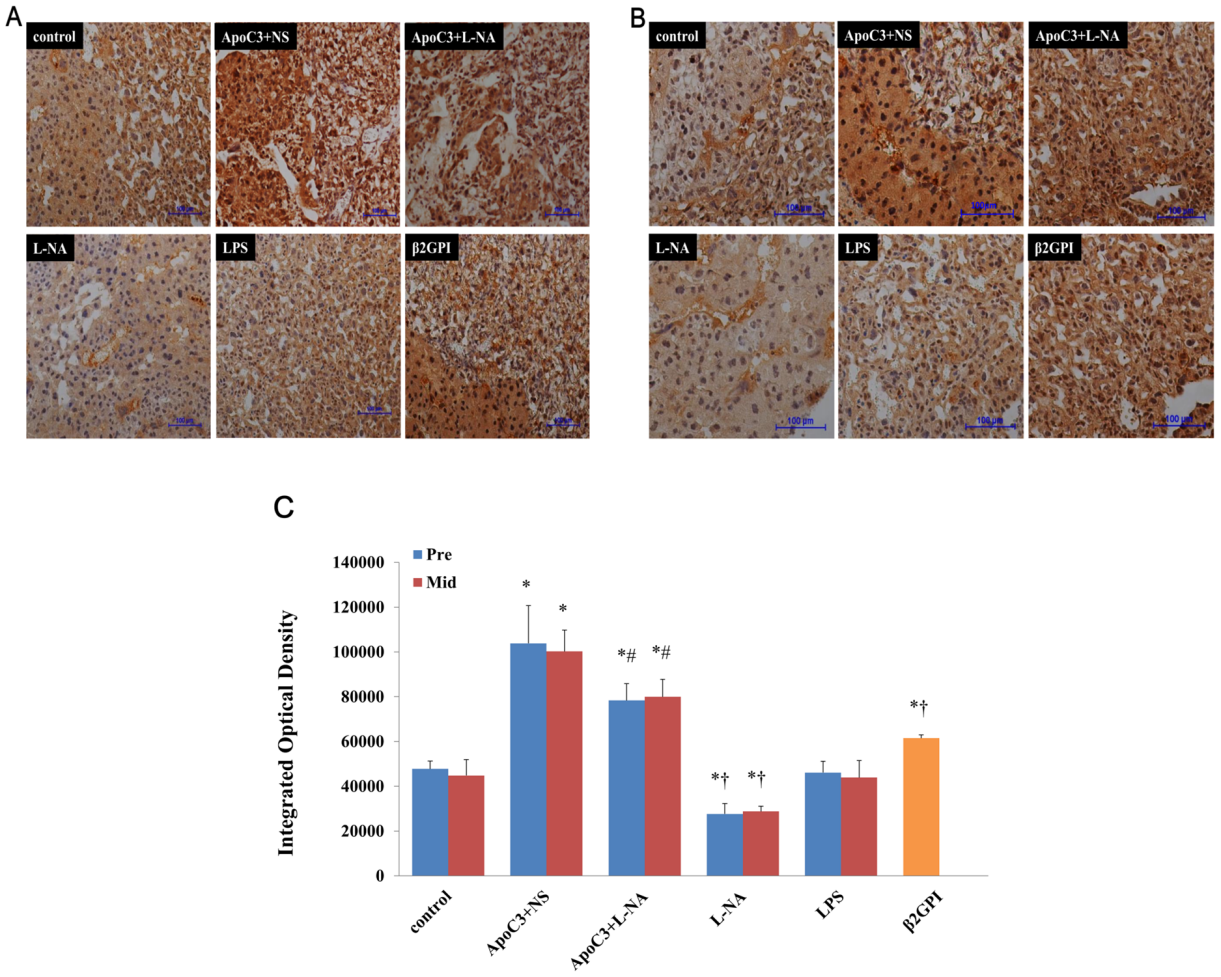
Representative immunohistochemical staining of LCHAD in mouse placenta at pre-implantation (A) and mid-gestational age (B). (Original magnification ×100, scale bars 100 µm) (C): IOD of placenta immunohistochemical images in all groups. *P<0.05 compared with control. †P<0.05 compared with ApoC3+L-NA. #P<0.05 compared with ApoC3+NS. Data are mean±SD, n = 10. Pre, pre-implantation. Mid, Mid-gestation.

Immunohistochemical staining showed LCHAD positivity in liver and placenta of pregnant mice in all groups (Fig. 6, 7). LCHAD expression was strongly positive for ApoC3+NS and ApoC3+L-NA mice and weakly positive for L-NA mice (Fig. 6C, 7C).

**Figure 8:**
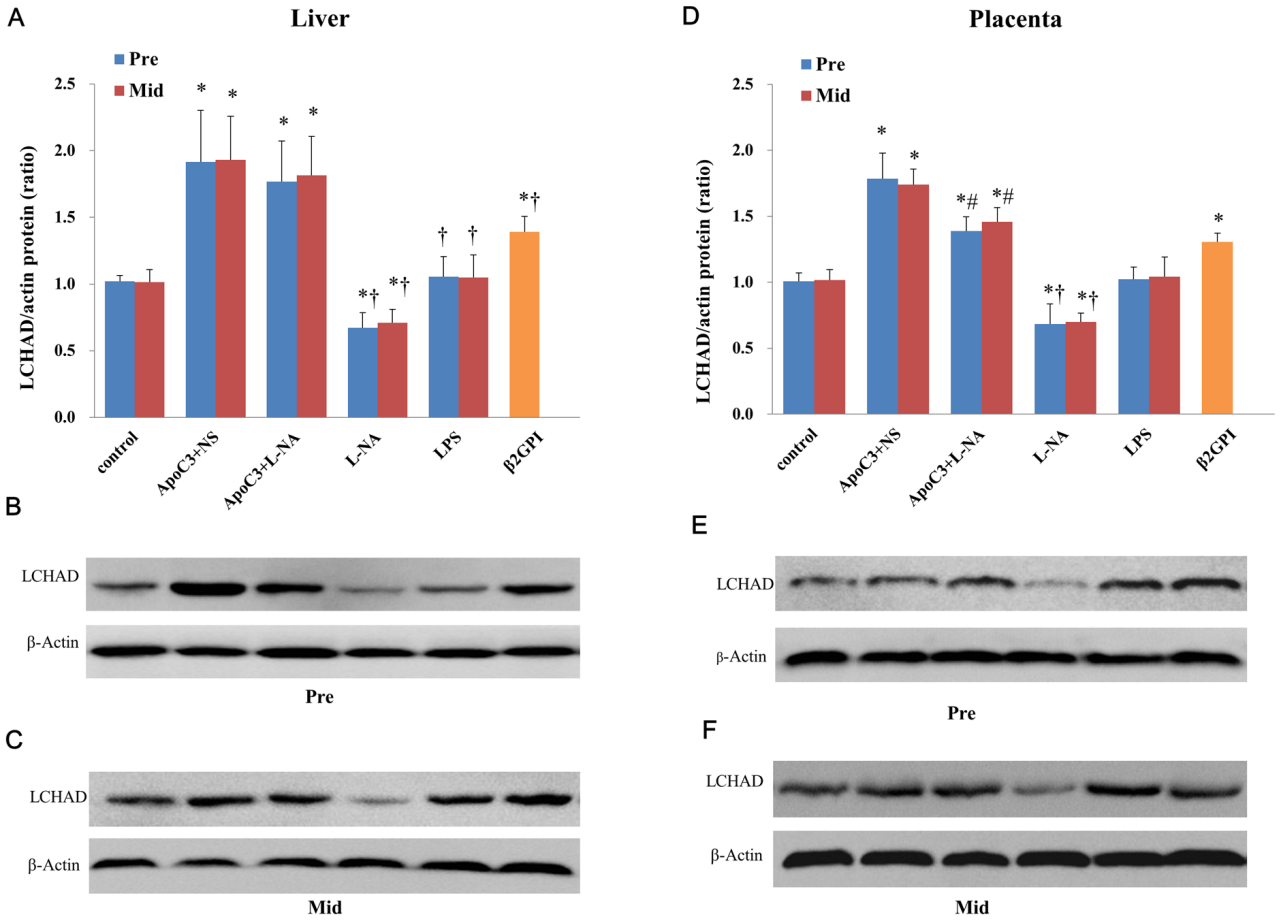
Western blot analysis and quantification of protein level of LCHAD in liver (A-C) and placenta (D-F). *P<0.05 compared with control. #P<0.05 compared with ApoC3+NS. †P<0.05 compared with ApoC3+L-NA. Data are mean±SD, n = 10. Pre, pre-implantation. Mid, Mid-gestation.

LCHAD protein expression in the liver (Fig. 8A) and placenta (Fig. 8D) did not differ between Pre and corresponding Mid subgroups. LCHAD protein levels in liver (Fig. 8A-C) and placenta (Fig. 8D-F) were significantly higher in ApoC3+NS, ApoC3+L-NA, and β2GPI mice; significantly lower in L-NA mice (P<0.05); and did not differ from controls for LPS mice. LCHAD protein expression in placenta was lower for ApoC3+L-NA than ApoC3+NS mice (P<0.05) (Fig. 8B).

### P47phox Mrna And Protein Expression In Liver And Placenta Tissues

**Figure 9:**
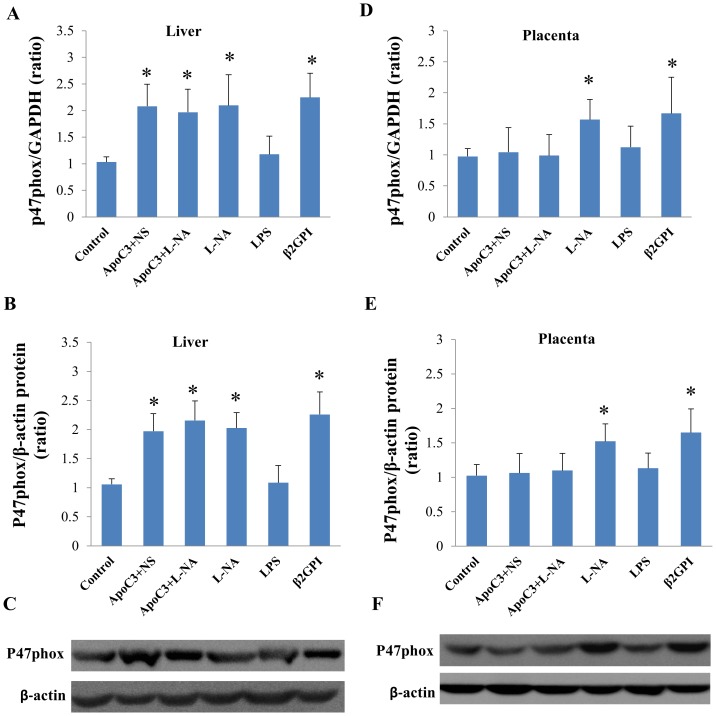
Quantified expression of p47phox mRNA and protein expression in liver (A-C) and placenta (D-F) in Mid groups. *P<0.05 compared with control. Data are mean±SD, n = 10.

We compared p47phox mRNA and protein expression in Mid groups and found significantly increased expression in the liver in all PE-like groups except the LPS group compared to the control group (P<0.05) (Fig. 9A-C). In the placenta, p47phox mRNA and protein expression significantly increased only in L-NA and β2GPI groups to control (P<0.05) (Fig. 9D-F).

### Correlation Between Ffa Levels, Lchad And P47phox Expression

We found FFA levels were significantly negatively correlated with LCHAD mRNA and protein expression in ApoC3+NS, ApoC3+L-NA and L-NA mice (P<0.05) and positively correlated in β2GPI mice in the liver (P<0.05), not correlated in LPS mice (Table S1). FFA levels were significantly positively correlated with p47phox mRNA and protein expression in the liver in all PE-like groups except the LPS group and significantly positively correlated in the placenta in L-NA and β2GPI mice (P<0.05) (Table S2). There was a significantly negative correlation between LCHAD and p47phox mRNA expression in the liver in ApoC3+NS mice and between LCHAD and p47phox protein expression in the liver in L-NA mice (P<0.05) (Table S3).

## Discussion

Numerous studies have explored the etiology, pathogenesis and treatment of PE by establishing different animal models with various methods [20]. Different PE-like animal models induced by different means support different theories and make a significant contribution to understanding this disease, but the interaction among multi-factorial PE needs more investigation.

Due to the difficulty of clinical specimen acquisition of different factor-induced PE patients, in this study we established four PE-like models for multi-factorial research of LCHAD expression changes to explore the effects of long-chain FAO in PE induced by different factors or different times. Treatments at both pre-implantation and mid-gestation could induce PE-like symptoms in pregnant mice.

L-NA and LPS treatment are classical methods for establishing PE-like models, and the LPS-established PE-like model mainly focuses on the mechanism of endothelial injury. However, most models started injection at the mid-gestation stage. Although PE symptoms appear after 20 weeks' gestation in humans, the abnormal placentation, trophoblast invasion and anomalous maternal-fetal immune interactions indicate that pathophysiologic features may exist at early gestation stage or even before pregnancy. So in addition to injection time at mid-gestation (day 11), we started injection before embryo implantation (day 3). Clinical findings suggest that APS is associated with PE, especially early-onset PE, and intrauterine death before 34 weeks [21]. To study changes of FAO in PE with maternal underlying disease, we established PE-like models associated with APS. ApoC3 transgenic mice show abnormal fatty acid metabolism, so we established PE-like models using these mice to observe the relationship between fatty acid metabolism and PE.

We found hypertension and proteinuria in the four PE-like models. Furthermore, after L-NA injection, ApoC3+L-NA mice showed PE-like symptoms, including hypertension and proteinuria, while ApoC3+NS mice showed hypertension alone. Animal and human studies have shown a strong positive correlation between plasma apoC3 and triglyceride concentrations, and ApoC3 transgenic mice show severe hypertriglyceridemia [22]. Hypertriglyceridemia is a risk factor for cardiovascular disease [23] and is associated with and precedes the onset of PE [24]. The results of this study suggested that hypertriglyceridemia might be a potential risk factor of hypertension. In addition, the APS model exhibited PE-like symptoms, which further indicated the relationship between maternal underlying disease and the pathogenesis of PE.

Blood pressure and urine protein was higher in Pre than Mid subgroups, which indicated that harmful factors in the early stage could aggravate PE clinical complications. The earlier the PE onset time is, the more severe the clinical complications are. Except for ApoC3+NS mice, the other PE-like groups showed adverse pregnancy outcomes. Placenta weight was lower in Pre than Mid subgroups in the L-NA group and LPS group, which further indicated that harmful factors in the early stage would aggravate PE clinical complications.

Although mice in ApoC3+NS group had abnormal fatty acid metabolism, lipid deposition in liver and placenta was lower for ApoC3+NS than other PE-like mice, which suggests that abnormal fatty acid metabolism alone is not sufficient to cause lipid deposition and PE-like symptoms. After L-NA injection, ApoC3+L-NA mice showed more lipid deposition in liver and placenta. LCHAD mRNA and protein expression in liver and placenta was higher for ApoC3+NS mice than controls, which suggests that high expression of the ApoC3 gene could increase FAO. After L-NA injection, LCHAD mRNA and protein expression in placenta was lower for ApoC3+L-NA than ApoC3+NS mice but still higher than for other groups. May be due to the increased serum FFA level in ApoC3 transgenic mice, FAO capacity was stimulated by long chain FFA resulting in LCHAD expression increased. High FFA levels will induce oxidative stress. In this study we found p47phox mRNA and protein expression in the liver increased in both ApoC3+NS and ApoC3+L-NA group and had positive correlation with FFA levels which proved the role of oxidative stress in the mechanism of abnormal FAO inducing PE.

L-NA is an inhibitor of endothelial nitric oxide synthase. Consistent with our previous study, LCHAD mRNA and protein expression was decreased with L-NA injection here. L-NA could induce high total cholesterol and TG levels in rat serum and decreased liver carnitine palmitoyltransferase activity, thus resulting in decreased FAO [25]. Also, L-NA could increase lipolysis by increasing lipolytic hormone secretion [26]. In this study, L-NA could decrease LCHAD mRNA and protein expression which resulting in increased FFA levels and p47phox expression. Thus we speculated that L-NA could increase lipolysis and decrease the activity of FAO enzymes and further induce accumulation of intermediate products and lipid deposition, which finally increased the occurrence of PE via lipotoxicity or combined action with oxidative stress. Mice in the LPS group showed lipid droplets in liver and placenta but significantly fewer than the other PE-like groups, with no significant difference in FFA levels, LCHAD and p47phox mRNA and protein expression compared to controls. Also, we found no correlation between FFA, LCHAD and p47phox expression in this group, which indicated that abnormal FAO may be not the significant mechanism in the pathogenesis of this type of PE. LPS may participate in the development of PE through other inflammation pathways. LPS can activate macrophagocytes to produce interleukin 1, 6 and tumor necrosis factor, which act on endothelial cells and activate an NF-κB inflammation pathway [27] and finally induce endothelial injury and PE-like symptoms.

Kajiwara and colleagues found that oxidized low-density lipoprotein (oxLDL) could combine with β2GPI to generate complexes easier for macrophage cytophagy [28], so lipid metabolism may play a role in the pathogenesis of APS. In this study, we found lipid droplets in liver and placenta of APS mice and increased FFA levels, which indicated abnormal lipid metabolism or FAO, but LCHAD mRNA and protein expression in liver and placenta was significantly increased compared to controls and positively correlated with FFA levels, so LCHAD might influence FAO through other pathways. The possible regulatory pathway of LCHAD in this PE-like model has been discussed in our last paper [29]. But in this model FAO disorders also participated in the pathogenesis of PE through oxidative stress.

The kinds of lipid droplets and their relationship with long-chain FAO in liver and placenta in LPS-treated mice are unclear and need further study. Lipid deposition in liver and placenta in LPS mice may be related to other abnormal lipid metabolism pathways other than long-chain FAO [30].

In conclusion, we have demonstrated that PE-like mouse models induced by different methods or different times of onset showed PE-like symptoms including hypertension, proteinuria, abnormal placenta and fetus development. LCHAD expression differed in different PE-like models. Long-chain FAO may play different role in different factorial PE and have a different cause-and-effect relation. FAO may interact with oxidative stress in the pathogenesis of PE. This work is beneficial to extending our understanding of the multi-factorial pathogenesis of PE and provides ideas for prediction and prevention of PE.

## Supporting Information

Table S1 **Correlation between FFA levels and LCHAD mRNA or protein expression in liver and placenta.**
(DOCX)

Table S2 **Correlation between FFA levels and p47phox mRNA or protein expression in liver and placenta.**
(DOCX)

Table S3 **Correlation between LCHAD mRNA or protein expression and p47phox mRNA or protein expression in liver and placenta.**
(DOCX)
