# Supplementary material for: Fatty Acid Oxidation Changes and the Correlation with Oxidative Stress in Different Preeclampsia-Like Mouse Models
Source: PLoS One. 2014 Oct 10;9(10):e109554. doi: 10.1371/journal.pone.0109554 (PMC4193787; doi:10.1371/journal.pone.0109554)
Supplement: Table S1 — (DOCX) [file pone.0109554.s001.docx]

**Table S1. Correlation between FFA levels and LCHAD mRNA or protein expression in liver and placenta.**

| **Groups** |  | **LCHAD mRNA** | | | | |  | **LCHAD protein** | | | | |
| --- | --- | --- | --- | --- | --- | --- | --- | --- | --- | --- | --- | --- |
|  | Liv: **r P** Pla: **r P** Liv: **r P** Pla: **r P** | | | | | | | | | | | |
| **ApoC3+NS** | -0.38 | | **0.0014** | -0.16 | 0.1888 | -0.35 | | | **0.0029** | -0.03 | 0.7814 |  |
| **ApoC3+L-NA** | -0.43 | | **0.0002** | -0.22 | 0.0683 | -0.37 | | | **0.0015** | -0.18 | 0.1161 |  |
| **L-NA** | -0.41 | | **0.0004** | -0.17 | 0.1576 | -0.43 | | | **0.0002** | -0.19 | 0.1217 |  |
| **LPS** | -0.22 | | 0.0672 | 0.15 | 0.2144 | -0.18 | | | 0.1202 | 0.16 | 0.6669 |  |
| **β2GPI** | 0.47 | | **0.0360** | 0.35 | 0.1282 | 0.50 | | | **0.0239** | 0.03 | 0.7918 |  |

Liv: liver. Pla: placenta.
