# Supplementary material for: Fatty Acid Oxidation Changes and the Correlation with Oxidative Stress in Different Preeclampsia-Like Mouse Models
Source: PLoS One. 2014 Oct 10;9(10):e109554. doi: 10.1371/journal.pone.0109554 (PMC4193787; doi:10.1371/journal.pone.0109554)
Supplement: Table S2 — (DOCX) [file pone.0109554.s002.docx]

**Table S2. Correlation between FFA levels and p47phox mRNA or protein expression in liver and placenta.**

| **Groups** |  | | **P47phox mRNA** | | | | |  | | **P47phhox protein** | | | |  |  |
| --- | --- | --- | --- | --- | --- | --- | --- | --- | --- | --- | --- | --- | --- | --- | --- |
|  | | Liv: **r P** Pla: **r P** Liv: **r P** Pla: **r P** | | | | | | | | | | | | | |
| **ApoC3+NS** | | | | 0.55 | **0.0002** | -0.04 | 0.8051 | | 0.42 | | **0.0072** | 0.10 | 0.5200 | |  |
| **ApoC3+L-NA** | | | | 0.76 | **0.0000** | 0.21 | 0.1874 | | 0.61 | | **0.0000** | 0.10 | 0.5478 | |  |
| **L-NA** | | | | 0.45 | **0.0038** | 0.43 | **0.0057** | | 0.42 | | **0.0073** | 0.42 | **0.0072** | |  |
| **LPS** | | | | 0.09 | 0.5714 | 0.01 | 0.9503 | | -0.28 | | 0.0777 | -0.07 | 0.6581 | |  |
| **β2GPI** | | | | 0.63 | **0.0030** | 0.72 | **0.0004** | | 0.45 | | **0.0471** | 0.45 | **0.0461** | |  |

Liv: liver. Pla: placenta.
