# Supplementary material for: Fatty Acid Oxidation Changes and the Correlation with Oxidative Stress in Different Preeclampsia-Like Mouse Models
Source: PLoS One. 2014 Oct 10;9(10):e109554. doi: 10.1371/journal.pone.0109554 (PMC4193787; doi:10.1371/journal.pone.0109554)
Supplement: Table S3 — (DOCX) [file pone.0109554.s003.docx]

**Table S3. Correlation between LCHAD mRNA or protein expression and p47phox mRNA or protein expression in liver and placenta.**

| **Groups** |  | | **P47phox mRNA** | | | |  | | **P47phhox protein** | | | | |
| --- | --- | --- | --- | --- | --- | --- | --- | --- | --- | --- | --- | --- | --- |
|  | | Liv: **r P** Pla: **r P** Liv: **r P** Pla: **r P** | | | | | | | | | | | |
| **ApoC3+NS** | | -0.32 | | **0.0454** | 0.18 | 0.2787 | | -0.17 | | 0.2842 | -0.02 | 0.9225 |  |
| **ApoC3+L-NA** | | -0.22 | | 0.1739 | 0.14 | 0.3919 | | 0.03 | | 0.8537 | -0.02 | 0.9116 |  |
| **L-NA** | | -0.13 | | 0.4134 | 0.19 | 0.2379 | | -0.37 | | **0.0174** | -0.08 | 0.6283 |  |
| **LPS** | | -0.01 | | 0.9375 | 0.18 | 0.2638 | | 0.10 | | 0.5197 | 0.16 | 0.3320 |  |
| **β2GPI** | | 0.16 | | 0.5052 | 0.20 | 0.3941 | | -0.12 | | 0.6160 | 0.09 | 0.7145 |  |

Liv: liver. Pla: placenta
